# Some Remarks on the Diagnosis and Complications of Diphtheria

**Published:** 1906-07-28

**Authors:** J. W. Miller

**Affiliations:** Assistant Medical Officer of Health, Blackburn.


### HOSPITAL CLINICS.

### SOME REMARKS ON THE DIAGNOSIS AND COMPLICATIONS OF DIPHTHERIA.

By J. W. MILLER, M.D. (Liverpool), D.Ph., Assistant Medical Officer of Health, Blackburn.

### POINTS IN DIAGNOSIS.

THE discovery of the diphtheria bacillus by Klebs in the membrane in the throat in cases of diphtheria, and the description of its growth on artificial media, as well as some of its pathogenic effects by Löffler in 1884, provided the means for the first time of a diagnosis being made by bacteriological examination in cases in which it is extremely difficult, and sometimes impossible, to diagnose a case by clinical means alone.

In the majority of cases a diagnosis can be made by clinical means. Along with the characteristic appearance of the throat, there are other symptoms which will assist in the diagnosis of this disease from other affections of the throat. In a typical case of diphtheria a high temperature is uncommon, and though the temperature may reach 103° F. during the first few days of the disease, more commonly it does not rise above 101 degrees, even when there is a large amount of swelling of the glands of the neck and extensive exudation; in fact, the severe forms of the disease are often less febrile than the milder forms in the former, the temperature often only reaching 97 or 98 degrees. Although the temperature is often low, usually the constitutional disturbance is well marked, and there is usually a fair amount of swelling of the glands of the neck.

In typical cases of the disease the diagnosis from follicular tonsillitis or pseudo-diphtheria caused by other organisms is not difficult, but cases do occur in which an accurate diagnosis can only be made by bacteriological methods.

*Simulation by Follicular Tonsillitis.*—Diphtheria may simulate cases of follicular tonsillitis, owing to the exudation which has filled the follicles of the tonsils spreading on to the adjacent surface; also in some cases of diphtheria the membrane, instead of commencing in the usual way at one spot and gradually extending, appears at several places on the tonsil at the same time, and produces an appearance like follicular tonsillitis. The temperature, constitutional disturbance, and swelling of the glands of the neck will assist in the diagnosis, in addition the presence of albumen in the urine. Albumen is present in the urine in about 50 to 60 per cent. of all cases; it may be only a trace or be in very large amount, associated with scanty urine. The albumen is present on about the third or fourth day of the disease, sometimes as early as the first day. It appears earlier and in larger amount in severe than in mild cases.

*Pseudo-diphtheria.*—The throat membrane may be caused by other organisms than the diphtheria bacillus, generally by streptococci, sometimes by pneumococci, and occasionally by blastomyces albicans. Microscopic examination of the membrane will show the characteristic appearance of the yeast in the last-mentioned case; in addition, the membrane is whiter, less adherent, and is usually present also on the buccal mucous membrane; it is seen occasionally in cases of enteric fever.

The membrane produced by streptococci is most commonly met with associated with scarlet fever. The membrane is of a more yellowish colour, is more friable, and less adherent than in diphtheria, and in scarlet fever is more often accompanied by some ulceration of the mucous membrane. Other symptoms associated with scarlet fever, such as the rash and tongue, will generally render the diagnosis clear, but diphtheria does sometimes occur along with scarlet fever in its early stages, and in all doubtful cases a swab should be taken for bacteriological examination.

*Catarrhal Tonsillitis.*—Bacteriological methods have proved that mild cases of diphtheria do sometimes occur indistinguishable from catarrhal tonsillitis. Such cases are seen during epidemics along with typical cases of the disease, and are also met with in association with well-marked cases of the disease in families.

The disease is often spread in schools either by means of mild unrecognised cases, children convalescent from the disease with bacilli still present in their throats, or children with bacilli in their throat unattended even by slight inflammation. I have seen diphtheria associated with scarlet fever in its early stages without the production of membrane. During 1904, out of 330 swabs taken from cases of scarlet fever on admission to the Blackburn Fever Hospital, I found diphtheria bacilli in two cases in which there was no membrane present in the throat. The occurrence of regurgitation on the taking of liquids, presence of a nasal voice, or other signs of paralysis, or the presence of croup during the first few weeks, have in certain cases first suggested the possibility of diphtheria along with scarlet fever; and in several cases I verified the diagnosis by examining a swab from the throat, the membrane in such cases may be absent or be present in so small an amount as to escape notice.

Regurgitation of fluids on swallowing is generally associated with severe angina in cases of scarlet fever, and occurs apart from the presence of diphtheria bacilli in the throat.

*Croup, Simple and Diphtheritic.*—Between simple and diphtheritic croup it is often impossible to make a diagnosis without a bacteriological examination. In association with measles or scarlet fever the diagnosis is usually clear, although in scarlet fever croup may be sometimes produced by diphtheria bacilli.

With simple catarrhal tonsillitis, especially if there is also present a sanguineous nasal discharge, diphtheria should be suspected.

It is especially important in doubtful cases that antitoxin should be injected immediately, before waiting for the result of the bacteriological examination, as such cases, if diphtheritic, progress very rapidly and the early injection of antitoxin may prevent the necessity for tracheotomy, and render the chances of recovery more certain.

*Diphtheritic rhinitis.*—This affection of the nose may occur primarily or in association with diphtheria of the fauces.

Occurring independently, it is apt to be overlooked. The discharge is thin and watery, or "mucopurulent," and in both cases is usually blood-stained. It is irritating, and generally causes some excoriation of the skin about the nose. In some cases epistaxis occurs, at times severe.

Occasionally membrane is seen, sometimes blocking up the whole of the anterior nares, but more commonly the membrane is obscured from being situated at the posterior part of the nose. On syringing, small pieces of membrane often come away, and sometimes a membranous cast of the whole interior of the nose, rendering the diagnosis clear. Associated with the nasal discharge you often get swelling and tenderness of the glands of the neck and albumen in the urine, whilst paralysis also sometimes occurs as in the other varieties of the disease.

There is often some constitutional disturbance, especially in young infants, but cases do occur in which there is neither any constitutional disturbance nor swelling of the glands of the neck. The character of the discharge will serve to distinguish diphtheritic rhinitis from simple coryza, but in any doubtful cases, especially of fibrinous rhinitis, a swab should be taken for bacteriological examination.

*Otorrhœa.*—This complication sometimes occurs associated with diphtheria, and the characteristic bacilli are found in the discharge. It is important that a patient with otorrhœa should be isolated so long as the discharge contains diphtheria bacilli. Diphtheritic lesions of the skin sometimes occur. There is ulceration of the skin, sometimes gangrenous, and often with a membranous slough covering the surface. Sometimes a membranous conjunctivitis occurs as the result of diphtheria bacilli. Where the ano-genital organs are affected there is usually diphtheria of the throat present at the same time.

In all doubtful cases a swab should be taken for bacteriological examination.

### Complications in Diphtheria.

*Paralysis.*—The more severe the attack of diphtheria and the younger the patient the more likely is paralysis to occur, the extent depending on the severity of the attack. Symptoms of paralysis generally appear about the second or third week, but sometimes as early as the fourth day—10 to 15 per cent. of all cases are affected. Paralysis may affect the palate alone. Attention is first drawn to it on account of regurgitation on swallowing, and often the presence of a nasal voice. When the paralysis extends it usually next affects the ciliary muscles of the eyeball, as shown by inability to accommodate for near objects; next the muscles of the eyeball, usually the external rectus are affected, indicated by squint. Sometimes the muscles of the eyeball or ciliary muscles are alone involved. The muscles next implicated are those of the lower limbs, followed by the muscles of the upper extremities and the respiratory muscles, including the diaphragm; in the worst cases of paralysis the laryngeal muscles are affected. Paralysis of the facial muscles and tongue are rare; occasionally paralysis is confined to the lower extremities.

Paralysis of the diaphragm generally comes on in association with paralysis elsewhere about the end of the fifth week, the paralysis being indicated by the abdomen being depressed instead of raised during inspiration, the reverse taking place during expiration. The muscles of the larynx affected are usually the adductors, loss of voice and ineffective cough resulting. When the palate is alone involved recovery usually takes place in a few weeks, and even when the paralysis is extensive recovery, usually complete, occurs in from six to eight weeks, although occasionally the paralysis lasts several months. Death may occur from asphyxia due to food getting into the larynx or from broncho-pneumonia from the same cause. With paralysis of the respiratory muscles, collection of mucus in the air passages takes place, and death occurs from this cause or from asphyxia, due to inability to expand the chest.

*Heart failure.*—In no disease are symptoms of heart failure of graver import than in diphtheria, a large percentage of deaths occurring from this cause.

In the early stages of the disease degenerative changes, chiefly fatty, are produced in the cardiac muscle, and changes are also produced in the motor nucleus of the vagus. Later in the disease there are less degenerative changes produced in the cardiac muscle, and more marked changes now occur in the nerve centres of the heart. The heart is affected to a greater or lesser extent in all cases of diphtheria; it is therefore important to watch carefully variations in the pulse from the commencement of the disease in mild as well as severe cases, as changes in the heart which would not be noticed in other diseases are of the greatest significance in diphtheria. If the pulse be examined carefully day by day, it will be found that irregularity is fairly common, although very slight in some cases, and occurs in mild as well as acute cases. Such irregularity is indicated by increased or decreased rate, generally the former and intermittence. In the early stages of the disease, in association with frequent vomiting and other signs of heart failure to be referred to presently, or in conjunction with scanty albuminous urine and repeated vomiting, irregularity of the pulse is of grave import. Irregularity of the pulse in the later stages is often noticed about the second or third week at the time when paralysis of the voluntary muscles first occurs, sometimes preceding this period, and also independently of paralysis elsewhere. The heart is affected to a greater extent the more extensive the paralysis. In the more severe cases of diphtheria signs of dilatation of the heart are soon perceptible, as shown by diffuse impulse, displacement of the apex beat downwards and outwards, and usually the presence of a systolic murmur at the apex, which disappears in the course of time. Endo- and peri-carditis are only occasional complications, and when present are generally caused by streptococci. In the most severe cases the heart takes some time to recover, and signs of dilatation can often be detected for seven or eight weeks after the onset of the disease.

Heart failure, though generally associated with severe cases, may occur in mild or moderate cases in which there has been a rapid disappearance of membrane, and where a complete recovery was expected. Failure of the heart usually occurs within the first fortnight, from about the fifth to the eighth day, but sometimes comes on as early as the second, and occasionally as late as the tenth, week. It is during the early stages that degenerative changes in the cardiac muscle are most marked, as at this time large quantities of toxins are being poured out into the circulation. Since the antitoxin treatment the number of deaths from heart failure has been considerably diminished as a definite neutralisation of the toxins by the antitoxin takes place. This most readily occurs during the first 48 hours, before an active combination of the toxins with the tissue cells has taken place. In cases which come under treatment on the fourth or fifth day or later, even when a large amount of antitoxin is injected, the action of the antitoxin is considerably diminished.

In the early stages of the disease, heart failure comes on gradually, being preceded generally by an irregular, intermittent pulse, and progresses slowly towards a fatal termination in the majority of cases. Sometimes death occurs suddenly owing to sudden collapse following an attack of vomiting, sitting up in bed, etc. In severe cases of laryngitis death may be accelerated through extra strain placed on the heart on account of difficult respiration.

Even when tracheotomy has relieved the breathing for the time being, collapse often follows after coughing up membrane or mucus. Associated with heart failure there is an irregular pulse and generally frequent vomiting. There is pallor, coldness of the extremities, and laboured breathing. The heart is dilated, and the pulse-rate is generally increased in frequency, reaching 140 to 170 beats a minute. Where slowing occurs the pulse-rate may fall to 20 a minute, and when this rate continues the prognosis becomes exceedingly grave. Frequent vomiting in diphtheria is always a grave symptom, being usually associated with heart failure; it also occurs in the early stages of the disease along with scanty urine containing a large quantity of albumen. In the later stages of the disease heart failure is usually associated with generalised paralysis. Although it sometimes occurs in cases where the palate, ciliary muscles, or muscles of the eyeball are alone involved, and sometimes independent of the paralysis of the voluntary muscles.

